# Identification of the APC/C co-factor FZR1 as a novel therapeutic target for multiple myeloma

**DOI:** 10.18632/oncotarget.12026

**Published:** 2016-09-15

**Authors:** Lisa J. Crawford, Gordon Anderson, Cliona K. Johnston, Alexandra E. Irvine

**Affiliations:** ^1^ Centre for Cancer Research and Cell Biology (CCRCB), Queen's University Belfast, Belfast, UK

**Keywords:** multiple myeloma, ubiquitin proteasome system, APC/C, FZR1, proTAME

## Abstract

Multiple Myeloma (MM) is a haematological neoplasm characterised by the clonal proliferation of malignant plasma cells in the bone marrow. The success of proteasome inhibitors in the treatment of MM has highlighted the importance of the ubiquitin proteasome system (UPS) in the pathogenesis of this disease. In this study, we analysed gene expression of UPS components to identify novel therapeutic targets within this pathway in MM. Here we demonstrate how this approach identified previously validated and novel therapeutic targets. In addition we show that FZR1 (Fzr), a cofactor of the multi-subunit E3 ligase complex anaphase-promoting complex/cyclosome (APC/C), represents a novel therapeutic target in myeloma. The APC/C associates independently with two cofactors, Fzr and Cdc20, to control cell cycle progression. We found high levels of FZR1 in MM primary cells and cell lines and demonstrate that expression is further increased on adhesion to bone marrow stromal cells (BMSCs). Specific knockdown of either FZR1 or CDC20 reduced viability and induced growth arrest of MM cell lines, and resulted in accumulation of APC/C^Fzr^ substrate Topoisomerase IIα (TOPIIα) or APC/C^Cdc20^ substrate Cyclin B. Similar effects were observed following treatment with proTAME, an inhibitor of both APC/C^Fzr^ and APC/C^Cdc20^. Combinations of proTAME with topoisomerase inhibitors, etoposide and doxorubicin, significantly increased cell death in MM cell lines and primary cells, particularly if TOPIIα levels were first increased through pre-treatment with proTAME. Similarly, combinations of proTAME with the microtubule inhibitor vincristine resulted in enhanced cell death. This study demonstrates the potential of targeting the APC/C and its cofactors as a therapeutic approach in MM.

## INTRODUCTION

Multiple Myeloma (MM) is a haematological neoplasm characterised by the clonal proliferation of malignant plasma cells in the bone marrow. Over the past decade, the introduction of new treatment strategies and novel agents such as proteasome inhibitors (PIs) and immuno-modulatory drugs (IMiDs), has significantly improved the clinical management of MM and extended overall survival [1, 2]. One of the most important therapeutic advances for MM has been the use of PIs to disrupt the ubiquitin proteasome system (UPS). Bortezomib, the first-in-class PI, is now a widely used component of MM therapy and a second generation PI, carfilzomib, was recently approved for the treatment of relapsed and refractory MM [3]. Despite the value of PIs to MM therapy, not all patients respond and acquired drug resistance [4] or dose-limiting side effects can limit their clinical utility [5]. This highlights the need to gain a better understanding of the molecular mechanisms of MM in order to develop additional treatment strategies.

The ubiquitin proteasome system (UPS) plays a central role in cellular protein homeostasis through the targeted destruction of damaged or mis-folded proteins and regulatory proteins that control many critical cellular processes. Protein degradation through this pathway occurs via two distinct and successive steps: ubiquitination of a target protein and subsequent degradation through the 26S proteasome. Target proteins are recruited for degradation through an enzymatic cascade involving three types of enzymes. An E1 (ubiquitin activating enzyme) activates ubiquitin and connects it to an E2 (ubiquitin conjugating enzyme); an E3 (ubiquitin ligase) then mediates the transfer of the activated ubiquitin to the target protein, thereby providing substrate specificity. The process can also be reversed by a group of proteases known as de-ubiquitinating enzymes (DUBs). Altered regulation of UPS components has been associated with transformation and tumorigenesis and therapeutic targeting of this pathway therefore holds great promise [6].

The success of PIs highlights the importance of the UPS in MM and it is becoming apparent that there are additional targets for therapeutic intervention in this pathway. E3 ligases and DUBs, in particular, present ideal therapeutic candidates as they may enable targeting of an aberrant protein or signalling pathway. Support for the potential of inhibiting these enzymes in MM comes from a number of studies. The first specific E3 ligase to be investigated as a therapeutic candidate was MDM2, an E3 ligase for the tumour suppressor p53. Small molecule inhibitors of MDM2, have been demonstrated to stabilize p53 and its substrates, resulting in apoptosis and cell cycle arrest in MM cell lines and primary cells [7, 8]. Small molecule inhibitors of the DUBs USP7, USP14 and UCHL5 have shown significant anti-MM activity, including in cells resistant to bortezomib [9, 10]. Indirect inhibition of a subset of E3 ligases known as cullin-RING E3 ligases (CRLs) using the NEDD8-activating enzyme (NAE) inhibitor MLN4924 has demonstrated encouraging activity in pre-clinical models of MM and recently completed a Phase 1 trial for patients with MM and lymphoma [11, 12]. Furthermore, IMiDs such as Thalidomide, have now been demonstrated to function, at least in part, by altering the activity of an E3 ligase complex [13].

We analysed gene expression of UPS components to identify novel therapeutic targets within this pathway in MM. This approach confirmed previously validated therapeutic targets and identified novel potential therapeutic targets. In this study, we show that FZR1, encoding for the protein fizzy-related protein homologue (Fzr, also known as Cdh1) represents a novel therapeutic target in MM. FZR1 is a cofactor of the multi-subunit E3 ligase complex anaphase-promoting complex/cyclosome (APC/C). The APC/C regulates cell proliferation by targeting multiple cell cycle regulators for ubiquitin-dependent degradation. Its activity depends on interaction with two main cofactors, Fzr and Cdc20, which interact transiently with the APC/C and select appropriate target proteins for ubiquitination. Cdc20 activates the APC/C during early mitosis while Fzr associates with the APC/C during late mitosis and promotes G1/S transition [14]. Chemical inhibition of the APC/C has been proposed as a therapeutic strategy in cancer [15–18] and has recently been reported to be an attractive potential therapy for MM [19]. The majority of these studies have explored the effects of APC/C inhibition on inhibiting APC/C^Cdc20^, while the role of APC/C^Fzr^ in tumorigenesis remains controversial. APC/C^Fzr^ has been reported as a tumour suppressor [20], however, two recent reports propose that altering levels of Fzr substrates through inhibition of the APC/C may also have therapeutic use. Eguren and colleagues identified topoisomerase IIα (TopIIα) as a protein substrate of Fzr and demonstrated that both knockdown of Fzr and inhibition of the APC/C with the inhibitor proTAME, significantly enhance sensitivity of tumour cells to TopIIα inhibitors [21]. Furthermore, inactivation of Fzr has been shown to result in replicative stress, cell cycle arrest and cell death, suggesting that APC/C^Fzr^ as well as APC/C^Cdc20^ are valid targets for anti-cancer therapy [22]. The aim of this study was to elucidate the importance and therapeutic potential of targeting APC/C^Fzr^ in MM.

## RESULTS

### Ubiquitin proteasome system gene expression signature in Multiple Myeloma

To identify a gene expression signature of UPS components in MM we initially performed topic-defined microarray analysis of 1286 UPS-related genes in U266 and OPM-2 cell lines compared to normal bone marrow (NBM) mononuclear cells (data available under GEO accession number GSE78884). Genes showing a ≥ 1.3-fold difference and a p-value of ≤ 0.05 were considered to be differentially expressed in the MM cell lines compared to NBM. Based on these criteria, there were 131 upregulated genes in OPM-2 and 173 upregulated genes in U266 cell lines, with an overlap of 75 genes; and 104 and 168 downregulated genes, respectively, with an overlap of 58 genes (Supplementary Figure S1A). Differentially expressed genes from our dataset were subsequently analyzed in 2 published datasets [23, 24] (GSE6691, GSE6477) to compare expression in CD138 selected cells from MM patients with CD138 selected cells from NBM; 47 genes showing a ≥ 2-fold difference and a p-value of ≤ 0.05 were selected for qPCR validation. Gene ontology analysis characterized this UPS gene set into 5 main subgroups; ubiquitination enzymes, deubiquitinating enzymes, proteasomal subunits, heat shock proteins and immune response-related. cDNA isolated from CD138 selected cells from NBM samples (n=3) and newly diagnosed and untreated MM patient samples (n=5), and from 4 MM cell lines (U266, OPM-2, KMS-18, JJN3) was analyzed on custom Taqman Low Density Arrays (TLDA) for the 47 genes. In general, there was good correlation with differentially expressed genes in TLDA arrays compared to the PIQOR array (r = 0.64; Supplementary Figure S1B) and 20 of the 47 genes were validated to have ≥ 2-fold change in expression across MM patient samples and cell lines when compared to normal CD138+ cells (Supplementary Figure S1C). Genes that were significantly increased in MM patient samples and cell lines versus normal CD138+ cells were found across all subgroups: ubiquitination enzymes (n=6), deubiquitination enzymes (n=2), proteasomal subunits (n=2), heat shock proteins (n=4) and immune response-related genes (n=2). Among these dysregulated genes there are a number that have already proven successful in pre-clinical and clinical investigations for MM therapy (Table 1). We have focused in this study on investigating the importance of FZR1, a cofactor of the APC/C E3 ligase complex, in MM.

**Table 1:** UPS-signature genes under pre-clinical or clinical investigation for MM

Gene	Function	Targeted Therapy	Reference
**BAG3**	Molecular chaperone	Cantharidin/norcantharidin	26–28
**HSPA8**	Heat shock protein 70	Ver-15508PET-16	29 30
**PSMB6**	Proteasome catalytic subunit β1	Bortezomib Carfilzomib	31,32
**USP7**	Deubiquitinase	P5091	9

### FZR1 is overexpressed in MM

**Figure 1:**
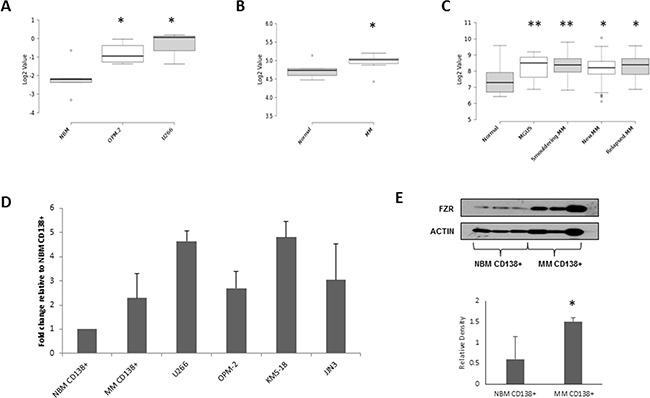
FZR1 expression levels in MM **A.** FZR1 gene expression in normal bone marrow (NBM ; n=4), OPM-2 (n=4) and U266 (n=3) MM cell lines; GSE78884. **B.** FZR1 gene expression in CD138+ cells from normal donors (n=5) and MM patients (n=12); data from published dataset GSE6477. **C.** FZR1 expression in CD138+ from normal donors (n=16), monoclonal gammopathy of undetermined significance (MGUS; n=22), smoldering MM (n=24), newly diagnosed MM (n=72) and relapsed MM (n=27); data from published dataset GSE6691. **D.** qPCR expression of FZR1 in CD138+ cells from healthy donors (n=3), MM patients (n=5) and 4 MM cell lines (U266, OPM-2, KMS-18, JJN3; n=3). **E.** Western blot analysis of FZR1 expression in CD138+ cells from NBM, MM patients and MM cell lines. Densitometry analysis is expressed as relative density corrected to loading control. * p ≤ 0.05, ** p ≤ 0.01 (t-test).

FZR1 mRNA expression was identified to be significantly higher (p ≤ 0.03) in U266 and OPM-2 cells compared to NBM in our data set (Figure 1A). Similarly, FZR1 expression was found to be significantly higher in CD138+ cells from MM patients compared to CD138+ cells from NBM in 2 independent patient datasets. Analysis of data from GSE6691 (Figure 1B) demonstrated increased expression in newly diagnosed MM patients (n=12; p = 0.01), while analysis of data from GSE6647 (Figure 1C) demonstrated a significant increase in FZR1 expression across 4 different stages of plasma cell dyscrasias - monoclonal gammopathy of undetermined significance (MGUS; n=22; p = 0.009), smoldering MM (n=24; p = 0.004), newly diagnosed MM (n=72; p = 0.02) and relapsed MM (n=27; p = 0.02). Analysis of publically available dataset GSE19784 [25] revealed that there was no significant difference in FZR1 expression across molecular subgroups of MM (Supplementary Figure S2A). Overexpression of FZR1 at the gene level was validated by qPCR analysis of CD138+ cells from MM patient samples and MM cell lines versus CD138+ cells from NBM (Figure 1D). Furthermore, Western blotting analysis using protein extracts from NBM CD138+ cells and MM CD138+ cells confirmed higher protein expression of Fzr in MM (Figure 1E). Analysis of the same datasets for APC/C cofactor CDC20 revealed that there was no significant difference in expression in MM compared to NBM (Supplementary Figure S2B-S2D), however, significantly higher CDC20 expression was observed across distinct molecular subgroups of MM (Supplementary Figure S2E).

### Knockdown of both FZR1 and CDC20 results in decreased viability and cell cycle arrest in MM cell lines

**Figure 2:**
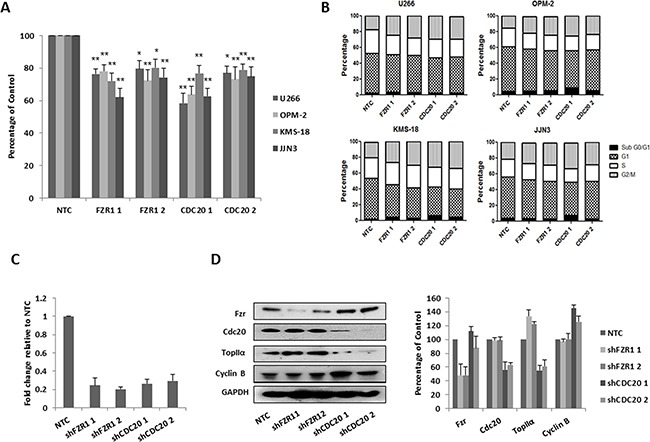
Knockdown of FZR1 and CDC20 reduces viability and induces cell cycle arrest **A.** MM cell lines were transduced with two different shRNA constructs against FZR1 or CDC20, viability of MM cell lines was measured 48 hrs post-puromycin selection. Results shown represent the mean of 3 independent experiments and are expressed as a percentage of cells transfected with non-targeting control (NTC) shRNA. **B.** Cell cycle analysis of propidium iodide stained cells 48 hrs post-transduction with shFZR1 or shCDC20. Results shown represent the mean of 3 independent experiments. **C.** q-PCR analysis of FZR1 and CDC20 gene expression in U266 cells 48 hrs post-transduction. Results shown represent the mean of 3 independent experiments and are expressed as fold-change relative to NTC. **D.** Protein expression of Fzr, Cdc20 and their respective substrates TopIIα and Cyclin B in U266 cells 48 hours post-transduction. Densitometry analysis is expressed as a percentage of NTC corrected to loading control. * p ≤ 0.05, ** p ≤ 0.01 (t-test).

APC/C inhibitor proTAME works by blocking the interaction of the APC/C with both Fzr and Cdc20 [33]. Therefore before analysis with this compound, we sought to first determine the effect of individual knockdown of either FZR1 or CDC20 on the growth and survival of MM cell lines. Cell lines were transduced with retrovirus encoding 2 distinct FZR1 and CDC20 – targeting shRNA constructs; cells transduced with a non-targeting control (NTC) shRNA were used as a control. Following puromycin selection, cells were seeded at a density of 3 x 10^5^ cells/ml and viability was evaluated after 48 hrs. Knockdown of either FZR1 or CDC20 resulted in a significant decrease (p ≤ 0.05) in viability of all cell lines (Figure 2A) and this was associated with an accumulation of cells in G2/M phase of the cell cycle (Figure 2B). Knockdown of FZR1 and CDC20 was confirmed at the gene (Figure 2C) and protein level (Figure 2D) and resulted in an increase in protein levels of APC/C^Fzr^ substrate TopIIα and APC/C^Cdc20^ substrate Cyclin B.

### Inhibition of APC/C^Fzr^ and APC/C^Cdc20^ with proTAME results in decreased viability and cell cycle arrest in MM cell lines

**Figure 3:**
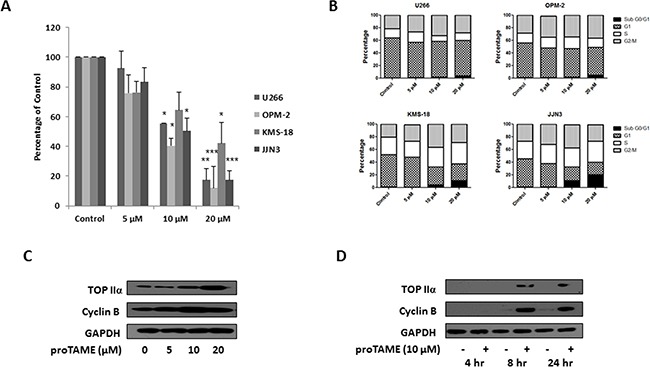
Inhibition of the APC/C^Fzr^ and APC/C^Cdc20^ in MM cell lines with proTAME **A.** Cell viability of MM cell lines 48 hrs after treatment with 5, 10 or 20 μM proTAME. Results are representative of 3 independent experiments and are expressed as a percentage of vehicle treated control. **B.** Cell cycle analysis of propidium iodide stained cells 48 hrs after treatment with 5, 10 or 20 μM proTAME. Results are representative of 3 independent experiments. **C.** Western blot analysis of TopIIα and Cyclin B levels in U266 cells 24 hrs after treatment with 5, 10 or 20 μM proTAME. **D.** Western blot analysis of TopIIα and Cyclin B levels in U266 cells 4, 8 or 24 hrs after treatment with 10 μM proTAME. * p ≤ 0.05, ** p ≤ 0.01, *** p ≤ 0.001 (t-test).

A recent study demonstrating that proTAME induced apoptosis and inhibited proliferation of MM cells primarily focused on its effects on blocking APC/C^Cdc20^ activity [19]. In this study we determined the effect of proTAME (5, 10, 20 μM) on the viability of MM cell lines, cell cycle and on expression of substrates of APC/C^Fzr^ and APC/C^Cdc20^. There was a dose-dependent decrease in viability of MM cell lines, with ≥ 35% reduction of viability observed following 24 hrs treatment with 10 μM proTAME (Figure 3A). Similar to knockdown of FZR1 or CDC20, we found that treatment with proTAME at lower doses (5 & 10 μM) led to an increase of cells in G2/M phase of the cell cycle, while a higher dose of proTAME (20 μM) led to an increase in the sub G0/G1 population, indicating induction of apoptosis (Figure 3B). An increase in the APC/C^Fzr^ substrate TopIIα and APC/C^Cdc20^ substrate Cyclin B was evident following treatment with 10 μM proTAME for 24 hrs. To identify when APC/C substrate accumulation was apparent, TopIIα and Cyclin B levels were evaluated 4, 8 and 24 hrs after treatment with 10 μM proTAME. An increase in both substrates was evident following 8 hrs treatment (Figure 3D) and this concentration and time-point were taken forward for use in combination studies.

### APC/C inhibition in combination with mitotic inhibitors significantly reduces viability of MM cell lines and primary MM cells

**Figure 4:**
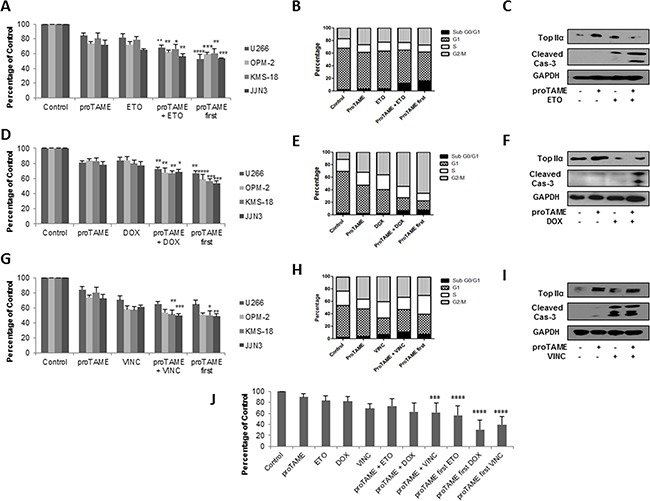
APC/C inhibition enhances the effect of conventional anti-mitotic agents in MM cell lines and primary cells **A-C.** MM cell lines were treated with 10 μM proTAME, 1μM etoposide (ETO), or a combination of both drugs added simultaneously or first pre-treated for 8 hrs with proTAME. **D-F.** MM cell lines were treated with10 μM proTAME, 100 nM doxorubicin (DOX), or a combination of both drugs added simultaneously or first pre-treated for 8 hrs with proTAME. **G-I.** MM cell lines were treated with10 μM proTAME, 10 μM vincristine (VINC), or a combination of both drugs added simultaneously or first pre-treated for 8 hrs with proTAME. (A,D,G) Cell viability 48 hrs post-treatment; results shown are the mean of 3 independent experiments and are expressed as a percentage of vehicle treated control. (B,E,H) Cell cycle analysis of U266 cells 48 hrs post-treatment. (C,F,I) Western blot analysis of TopIIα and cleaved caspase-3 48 hrs post-treatment. **J.** CD138+ cells from 3 MM patients were treated with 10 μM proTAME, 1 μM ETO, 100 nM DOX or 10 μM VINC as single agents or a combination of proTAME with each drug added simultaneously or first pre-treated for 8 hrs with proTAME. * p ≤ 0.05, ** p ≤ 0.01, *** p ≤ 0.001, **** p ≤ 0.0001 (one-way ANOVA).

Knockdown of FZR1 or inhibition of the APC/C with proTAME has been reported to enhance the sensitivity of a number of tumour cell lines to the topoisomerase II inhibitor etoposide, through an increase in APC/C^Fzr^ substrate TopIIα [21]. We therefore evaluated the effect of proTAME (10 μM) or FZR1 knockdown in combination with etoposide (1 μM) and doxorubicin (100 nM), a commonly used topoisomerase II inhibitor in MM therapy. ProTAME was combined with the topoisomerase inhibitors either by adding both drugs simultaneously or by first treating cells with proTAME for 8 hrs before adding either etoposide or doxorubicin. Simultaneous combination with proTAME resulted in significantly reduced cell viability compared to single agent alone (Figure 4A & 4D), and similar results were replicated when etoposide or doxorubicin were combined with FZR1 knockdown (Supplementary Figure S3A & S3B). More pronounced effects on cell viability were seen if cells were first pre-treated with proTAME. Cell cycle analysis demonstrated that etoposide as a single agent increased the percentage of cells in G2/M and S phase, while doxorubicin led to a significant increase in G2/M. Combination of both topoisomerase inhibitors with proTAME resulted in an increase in the sub G0/G1 population (Figure 4B & 4E and Supplementary Figure S4A & S4B). Consistent with this Western blot analysis demonstrated an increase in cleaved caspase-3, indicating induction of apoptosis, when proTAME or FZR1 knockdown was combined with either drug. TopIIα levels were shown to increase following treatment with proTAME or FZR1 knockdown, and this was abrogated following treatment with etoposide or doxorubicin in combination with proTAME (Figure 4C & 4F and Supplementary Figure S3D & S3E).

Microtubule inhibitors indirectly result in inhibition of APC/C activity and it has been proposed that combination of microtubule inhibitors with an APC/C inhibitor may enhance cell death [33]. We therefore investigated the combination of proTAME or FZR1 knockdown with the microtubule inhibitor vincristine in MM cell lines. As above, the drugs were combined both simultaneously or first pre-treated with proTAME for 8 hrs. The combination of proTAME (10 μM) or FZR1 knockdown with vincristine (10 μM) resulted in significantly reduced viability in 2 out of 4 cell lines compared to either agent alone, pre-treatment with proTAME did not enhance cell death any further (Figure 4G and Supplementary Figure S3). Cell cycle analysis demonstrated that vincristine as a single agent increased the percentage of cells in G2/M and sub G0/G1 populations. Combination with proTAME led to a reduction of cells in G2/M phase but no further increase in sub G0/G1 phase (Figure 4H and Supplementary Figure S4C). Western blot analysis demonstrated a similar increase in cleaved caspase-3 both with vincristine as a single agent and in combination with proTAME or FZR1 knockdown. TopIIα levels, as expected, did not change following treatment with vincristine (Figure 4I and Supplementary Figure S3F).

Proteasome inhibitors (PIs), immunomodulatory agents (IMiDs) and corticosteroids form the backbone of many current myeloma treatment schedules, therefore, we also investigated the effects of proTAME in combination with 10 nM carfilzomib (PI), 20 μM pomalidomide (IMiD) or 20 nM dexamethasone (corticosteroid). There was limited benefit observed to combining proTAME with any of these agents (Supplementary Figure S5).

CD138+ cells were selected from 3 MM patient BM aspirates and were treated with proTAME in combination with etoposide, doxorubicin or vincristine using the same conditions as before. Figure 4J demonstrates that pre-treatment with proTAME resulted in a highly significant reduction in viability for all three drugs compared to single agents, and the simultaneous combination of proTAME with doxorubicin and vincristine demonstrated a significant benefit in primary cells.

### BMSC adhesion results in increased Fzr expression

**Figure 5:**
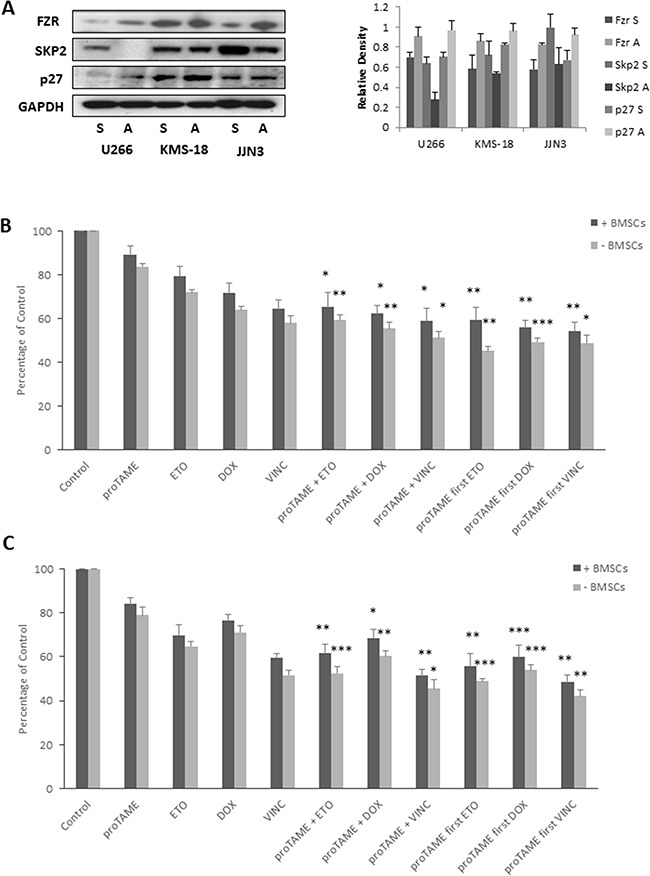
BMSC adhesion results in increased Fzr expression **A.** Fzr, Skp2 and p27 protein expression in U266, KMS-18 and JJN3 cells cultured in suspension (S) or adhered (A) to HS-5 stromal cell line. Densitometry analysis is expressed relative to loading control. **B** & **C.** U266 (B) and JJN3 (C) grown with and without primary BMSCs from 3 different MM patients and treated with 10 μM proTAME in combination with 1 μM etoposide (ETO), 100 nM doxorubicin (DOX) or 10 μM vincristine (VINC). Drugs were combined either simultaneously or first pre-treated with proTAME for 8 hrs. Proliferation was assessed 48 hrs post-treatment and results are expressed as a percentage of vehicle treated control. * p ≤ 0.05, ** p ≤ 0.01, *** p ≤ 0.001 (one-way ANOVA).

BMSCs are well documented to play an important role in the survival of MM cells and contribute to cell adhesion - mediated drug resistance. Lwin and colleagues previously reported that adhesion of lymphoma cell lines to BMSCs causes the cells to enter a protected quiescent state, mediated through upregulation of Fzr, leading to degradation of APC/C^Fzr^ substrate Skp2 and an accumulation of p27 [34]. To determine if cell adhesion affects Fzr expression in MM, we co-cultured MM cell lines with the stromal cell line HS-5 for 24 hrs, detached adherent MM cells from stromal cells and analyzed protein expression alongside cells grown in suspension. Similar to the findings by Lwin and colleagues we found an increase in Fzr protein expression in MM cell lines that were adhered to HS-5 cells, and a reciprocal decrease in Skp2 and increase in p27 expression (Figure 5A). As BMSCs can protect against drug cytotoxicity, we investigated the effect of proTAME in combination with etoposide, doxorubicin or vincristine on MM cells co-cultured with or without BMSCs derived from 3 individual MM patient samples. The combination of proTAME with all 3 compounds results in a significant (p ≤ 0.05) decrease in viability, even when cultured in the presence of BMSCs (Figure 5B–5C).

## DISCUSSION

The introduction of novel treatment strategies for MM, including the use of PIs bortezomib and carfilzomib has led to improved response rates and overall survival and highlighted the importance of the UPS in this malignancy. However, the majority of MM patients will ultimately relapse or develop refractory disease, emphasizing the need for the identification of additional therapies. The UPS consists of many components, most of which have the potential to be pharmacologically targeted. With this in mind we sought to generate a gene expression signature of UPS-related genes in MM to identify novel potential therapeutic targets. Through the use of UPS topic-defined microarray analysis of MM cell lines, combined with interrogation of published MM patient datasets, we identified 20 UPS-related genes with differential expression in MM compared to normal CD138+ plasma cells. Four of these genes (BAG3, HSPA8, PSMB6, USP7) are targeted by inhibitors already in clinical or pre-clinical studies for MM, giving confidence to the ability of this approach to identify UPS-related therapeutic targets in MM. Given the interest in the potential of targeting the APC/C in cancer, we selected APC/C cofactor Fzr from this signature to bring forward for further investigation in this study.

The APC/C is an important E3 ligase complex involved primarily in cell cycle regulation and its activity is dependent on association with 2 cofactors, Cdc20 and Fzr. While Cdc20 is known to target a limited number of important mitotic regulators for degradation, Fzr is much more promiscuous [21]. APC/C^Cdc20^ plays an essential role in the degradation of Cyclin B and securin and thus, inhibition of the APC/C by preventing Cdc20-dependent mitotic exit has been the main focus of therapeutic approaches to targeting the APC/C. To date little has been studied on the effect of the associated inhibition of the APC/C^Fzr^ by APC/C inhibitor proTAME. Indeed it may even have been thought of as an undesired side effect, based on reports of the tumour suppressor effects of Fzr in some tumour types. However, accumulating evidence suggests that targeting the APC/C^Fzr^ may also be of benefit to anti-cancer therapies. Based on our gene expression array data combined with 2 published patient datasets, we found significantly higher FZR1 gene expression in MM compared to NBM. This is in agreement with a previous study comparing gene expression in plasma cells from a MM patient with plasma cells from a genetically identical twin, in which up-regulation of FZR1 was identified [35]. MM is preceded by pre-malignant conditions, MGUS or smoldering myeloma, which progress to malignant MM at a rate of 1% and 10% per year, respectively [36]. Interrogation of dataset GSE6647 demonstrates that FZR1 expression is also upregulated in these precursor diseases, suggesting that dysregulation of FZR1 expression may occur early in the disease. Consistent with Lub and colleagues [19], we found that FZR1 expression does not appear to be associated with any particular cytogenetic or risk group as no difference in expression was observed across molecular subtypes of the disease. CDC20 expression on the other hand did not differ significantly in MM compared to NBM in any of the datasets analyzed in this study, however, again in agreement with Lub and colleagues we did observe higher levels of CDC20 expression in certain molecular subtypes.

Prior to examining the effect of proTAME on both APC/C^Cdc20^ and APC/C^Fzr^ in MM, we first looked at the effect of genetic knockdown of each cofactor individually. Knockdown of either Fzr or Cdc20 resulted in a comparable reduction in viability of MM cell lines and a G2/M arrest. These results are consistent with a number of studies in MM and other cancers that have investigated the consequence of Cdc20 knockdown [19, 37, 38]. Furthermore, similar effects on the cell cycle and cell death have previously been reported following knockdown of Fzr [22, 39] supporting the potential of Fzr, as well as Cdc20 as a therapeutic target in MM. It was recently suggested that proTAME may preferentially inhibit APC/C^Cdc20^ in MM [19]. In this study, we observed an equal accumulation of an APC/C^Fzr^ substrate (TopIIα) and an APC/C^Cdc20^ substrate (Cyclin B) following proTAME treatment, which is in accordance with earlier studies demonstrating that proTAME inhibits the interaction of both cofactors [33]. A dose-dependent decrease in viability was seen with proTAME in the cell lines and consistent with our knockdown studies, and previous studies, we found that proTAME induced a G2/M arrest leading to an increase in apoptosis with higher doses.

Following the identification of TopIIα as a substrate of APC/C^Fzr^, Eguren and colleagues found that the combination of proTAME (to inhibit Fzr) and etoposide (to inhibit TopIIα) displayed a synergistic effect in tumour cells [21]. Correspondingly, we found that both proTAME and knockdown of FZR1 significantly enhanced the anti-proliferative effect of two topoisomerase inhibitors in MM cell lines and primary cells. We also investigated the combination of proTAME with the microtubule inhibitor vincristine in MM. Microtubule inhibitors disrupt the mitotic spindle, leading to activation of the spindle assembly checkpoint (SAC) and inhibition of APC/C. Although microtubule inhibitors are an important class of anti-tumour agent, the response of tumour cells to these agents can be highly variable. Zeng and colleagues, postulate that as the SAC does not completely supress APC/C activity that the cells rely on continued protein synthesis to maintain mitotic arrest [33]. Therefore they suggest that combination of proTAME and a microtubule inhibitor may lead to sustained mitotic arrest and enhanced cell death. Our findings support this theory and show that the combined treatment of proTAME or FZR1 knockdown with vincristine results in enhanced cell death in MM. Although proTAME in combination with a PI, IMiD or corticosteroid did not enhance cell death, doxorubicin, and to a lesser extent vincristine, are currently used in combination with novel therapies for MM [40], demonstrating that proTAME can enhance the activity of clinically relevant MM therapies.

MM cells reside and proliferate almost exclusively in the BM and the BM microenvironment plays a well-established role in supporting MM cell growth and survival [41]. MM cells adhere to BMSCs in the BM resulting in the activation of many pathways promoting growth and survival of MM cells and protecting cells from drug-induced apoptosis. Using a mantle cell lymphoma (MCL) model, Lwin and colleagues demonstrated that adhesion of MCL cells to BMSCs resulted in a reversible growth arrest that correlated with a decrease in Skp2 and an increase in p27 levels in the MCL cells [34]. Moreover, they found that BMSC adhesion upregulated Fzr expression and that APC/C^Fzr^ was an upstream effector of the Skp2/p27 signalling pathway. As adhesion to BMSCs has previously been found to induce a similar reversible growth arrest in MM cells [41, 42], we sought to investigate Fzr levels in MM cells upon adhesion to BMSCs. In this study, we show a similar upregulation of Fzr in MM cells and associated decrease in Skp2 and increase in p27 expression. These observations imply that this may be a mechanism by which BMSCs help to protect malignant MM cells against drug-induced apoptosis. In support of this theory, the APC/C was recently implicated in cancer cell dormancy and protection against drug-induced apoptosis in lung cancer cell lines [43]. We furthermore investigated the effect of proTAME in combination with etoposide, doxorubicin or vincristine in MM cell lines co-cultured with MM patient-derived BMSCs, which are known to support the survival of MM cells to a greater extent than normal BMSCs [44]. The combination of proTAME and the conventional anti-mitotic agents still resulted in a significantly enhanced reduction in cell viability in the presence of BMSCs suggesting that these combinations could target MM cells in the BM microenvironment. Interestingly, Fzr has also been shown to regulate osteoblast function through modulation of Smurf1 activity and depletion of Fzr has been found to induce osteoblast differentiation through this pathway [45]. Suppression of osteoblast activity plays a key role in bone destruction and disease progression in MM [46]. The success of bortezomib in MM therapy can be partially attributed to its ability to promote osteoblast differentiation in MM [47] and with this is mind, the effects of Fzr on osteoblast function in the MM setting warrants investigation.

In summary, our studies identified a number of UPS components with dysregulated expression in MM, some of which have been previously validated. We have demonstrated that the APC/C cofactor Fzr is highly expressed in MM and expression increases further on adhesion to BMSCs. While many studies to date focus on blocking the activity of APC/C^Cdc20^ as an anti-cancer strategy, we show that APC/C^Fzr^ also represents an attractive potential therapeutic target in MM. Furthermore, the combination of APC/C^Fzr^ and APC/C^Cdc20^ inhibition with clinically relevant anti-mitotic agents results in enhanced anti-MM activity, even in the presence of BMSCs. The APC/C represents a promising candidate for further preclinical investigation in MM.

## MATERIALS AND METHODS

### Reagents

ProTAME was purchased from Boston Biochem (Cambridge, MA) as a 20 mM stock solution in DMSO, stored at −20°C and diluted in RPMI 1640 (Fisher Scientific, Loughborough, UK) immediately before use. Etoposide, doxorubicin and vincristine were purchased from Cayman Chemical (Cambridge, UK), reconstituted in DMSO and stored at −20°C.

### Cell lines

U266, OPM-2, KMS-18, JJN3, HS-5 and Phoenix GP cells were cultured in a humidified incubator at 37°C and 5% CO_2_ in RPMI 1640 medium supplemented with 10% fetal bovine serum and 100 U/mL penicillin, 100 μg/mL streptomycin (Fisher Scientific, Loughborough, UK). Authentication using short tandem repeat profiling (STR) was conducted by LGC Standards Cell Line Authentication Service within 1 year of use for these experiments.

### Primary samples

Bone marrow samples from MM patients were obtained with ethical approval from the Northern Ireland Biobank (approval # NIB12-0061) and those involved gave their informed consent in accordance with the Declaration of Helsinki. Human bone marrow mononuclear cells were purchased from Stem Cell Technologies (Cambridge, UK) for use as a normal control. CD138 expression is a hallmark of plasma cells and multiple myeloma cells. CD138+ cells were enriched from primary samples using the AutoMACS system (Miltenyi Biotech, Germany) according to the manufacturer's instructions.

### PIQOR ubiquitin-proteasome pathway gene expression array

Topic defined PIQOR (Parallel Identification and Quantification Of RNAs) Microarrays (Miltenyi Biotech, Germany) comprising 1286 UPS-associated genes were used to generate gene expression profiles of normal bone marrow (NBM) versus MM cell lines. mRNA was isolated from NBM (n=5) and MM cell lines [U266 (n=3) and OPM-2 (n=5)] and cDNA synthesized using the μMACS™ One-step T7 Template Kit according to the manufacturer's instructions (Miltenyi Biotech, Germany). Cy3-labelled samples of interest were hybridized against a Cy5-labelled common reference according to the manufacturer's instructions (Miltenyi Biotech, Germany). Microarray slides were scanned with an Axon GenePix 4400A microarray scanner (MDS analytical technologies) and fluorescence intensity data were measured by GenePix Pro 7.0. Raw data was background corrected (normexp), normalized within each array with a modification of the LOWESS method (print-tip loess normalization) and between all arrays (quantile normalization) with the R/Bioconductor package Limma. The medians of the log2 normalized intensities for each array, or the log2-ratios relative to the co-hybridized reference RNA were subsequently calculated for each probe set from the four replicate values. For statistical group comparisons between OPM or U266 cells and NBM cells, a two-sided t-test with equal variance was used (R/Bioconductor) using the normalized log2-ratio data.

### Taqman low-density array

Total RNA was isolated from cells using Trizol (Life Technogies, Paisley, UK), and converted to cDNA using MMLV (Life Technologies Paisley, UK) according to the manufacturer's instructions. Custom TaqMan® Array plates were designed to validate gene expression arrays of 48 UPS associated genes. Cycling conditions were as follows; 40 cycles of 95°C for 15 sec, followed by 60°C for 1 min. Each array was analysed on the 7900 system using Taqman Low Density Array (TLDA) default thermal-cycling conditions and 18S used as the endogenous control (Applied Biosystems, Paisley, UK).

### Retroviral transfection

Retroviruses expressing an shRNA against FZR1 and CDC20 were generated by transfection of a pRSC vector together with pMD2.G into Phoenix GP cells. shRNA sequences are shown below. Cells infected with vector expressing non-targeting control (NTC) shRNA were used as controls. Cells were selected in 0.5 μg/ml puromycin (Sigma-Aldrich, Dorset, UK), 48 hrs post-transduction.

shFZR1 1 CCCGTTCGACAAAGGTAAA

shFZR1 2 TGAGAACACGATGCCACGC

shCDC20 1 CGGCTTCGAAATATGACCA

shCDC20 2 GGTTCAGACCACTCCTAGC

### Cell viability

Cell Titer-Glo® luminescent assay (Promega, Southampton, UK) was used to analyze cell viability by measuring ATP levels according to the manufacturer's instructions. Luminescence was measured using a Tecan Genios microplate reader.

### Bone marrow stromal cell (BMSC) culture and MM co-culture assay

BM aspirates from MM patients were subjected to mononuclear cell separation by Ficoll-Hipaque density sedimentation, CD138 depleted and cultured *in vitro* for at least 3 weeks to establish long-term BMSC cultures. The adherent cell monolayer was harvested in HBSS containing 0.25% trypsin and 0.02% EDTA (Fisher Scientific, Loughborough, UK), washed, and collected by centrifugation. MM cell lines or MM patient-BMSCs were cultured either alone or together at 1:5 (BMSC/MM) ratio for 48 hrs, and cell proliferation was measured using the non-radioactive WST-1 colorimetric assay, as per manufacturers' instructions (Roche, Sussex, UK).

### Cell cycle analysis

Cells were harvested, washed in phosphate-buffered saline and fixed in 70% ethanol. Fixed cells were stained with 50 μg/ml propidium iodide solution containing 0.25 mg/ml RNase. DNA content was measured with an LSRII flow cytometer and subpopulations were identified using FACS Diva and Flowing Software (Turku Centre for Biotechnology, Finland).

### Western blotting

Cells were harvested and lysed in radioimmuno precipitation assay buffer containing protease and phosphatase inhibitors. Equal amounts of protein were denatured in LDS sample buffer (Invitrogen Ltd, Paisley, UK) at 95°C for 5 minutes, resolved by SDS-PAGE on 10% Bis-Tris gels (Invitrogen Ltd, Paisley, UK) and subsequently transferred to a polyvinylidene fluoride membrane. Immunoblotting was carried out using antibodies against *FZR1*, Topoisomerase II α, GAPDH (Abcam, Cambridge, UK), Pan-Actin, Cyclin B, Cleaved Caspase-3, SKP2, p27 (Cell Signaling Technology, Hertfordshire, UK) and CDC20 (Santa Cruz, Heidelberg, Germany) and secondary antibodies anti-mouse and anti-rabbit (DAKO, Cambridgeshire, UK). Blots were scanned into the AutoChemi System (UVP, Cambridge, UK) and analysed using LabWorks 4.5 image acquisition and analysis software.

## SUPPLEMENTARY FIGURES


