# Supplementary material for: Identification of the APC/C co-factor FZR1 as a novel therapeutic target for multiple myeloma
Source: Oncotarget. 2016 Sep 15;7(43):70481–93. doi: 10.18632/oncotarget.12026 (PMC5342567; doi:10.18632/oncotarget.12026)
Supplement: Supplementary file 1 [file oncotarget-07-70481-s001.pdf]

Identification of the APC/C co-factor FZR1 as a novel therapeutic target for multiple myeloma

SUPPLEMENTARY FIGURES

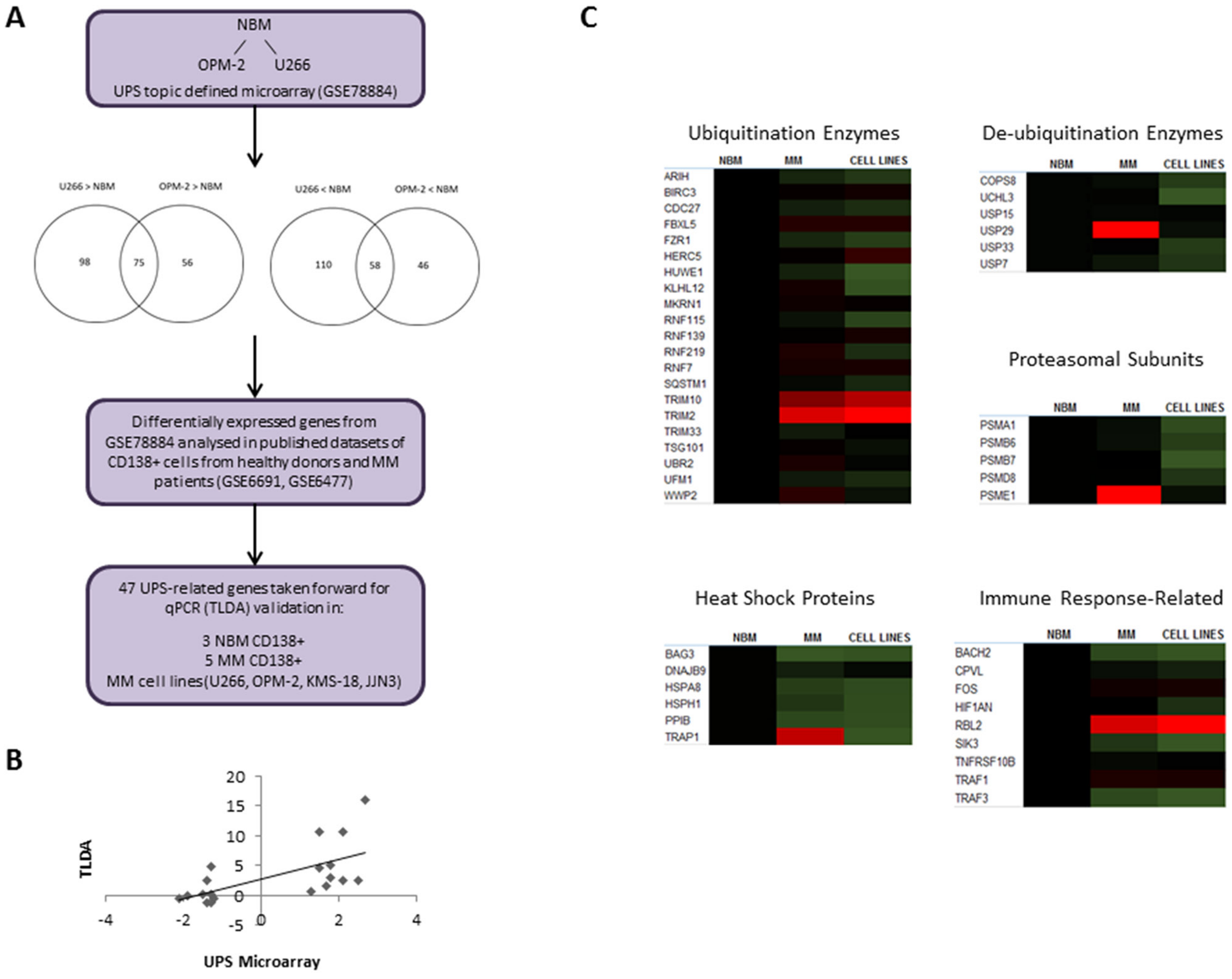

Supplementary Figure S1: A. Schematic of UPS gene signature identification. **B.** Correlation of gene expression in PIQOR microarrays and TLDA q-PCR validation. **C.** Heatmap of gene expression in CD138+ cells from MM patient samples (n=5) and MM cell lines (U266, OPM-2, KMS-18, JJN3) compared to CD138+ cells from NBM (n=3). Results are expressed as fold change relative to NBM CD138+ cells, Red < 1, Black =1, Green >1.

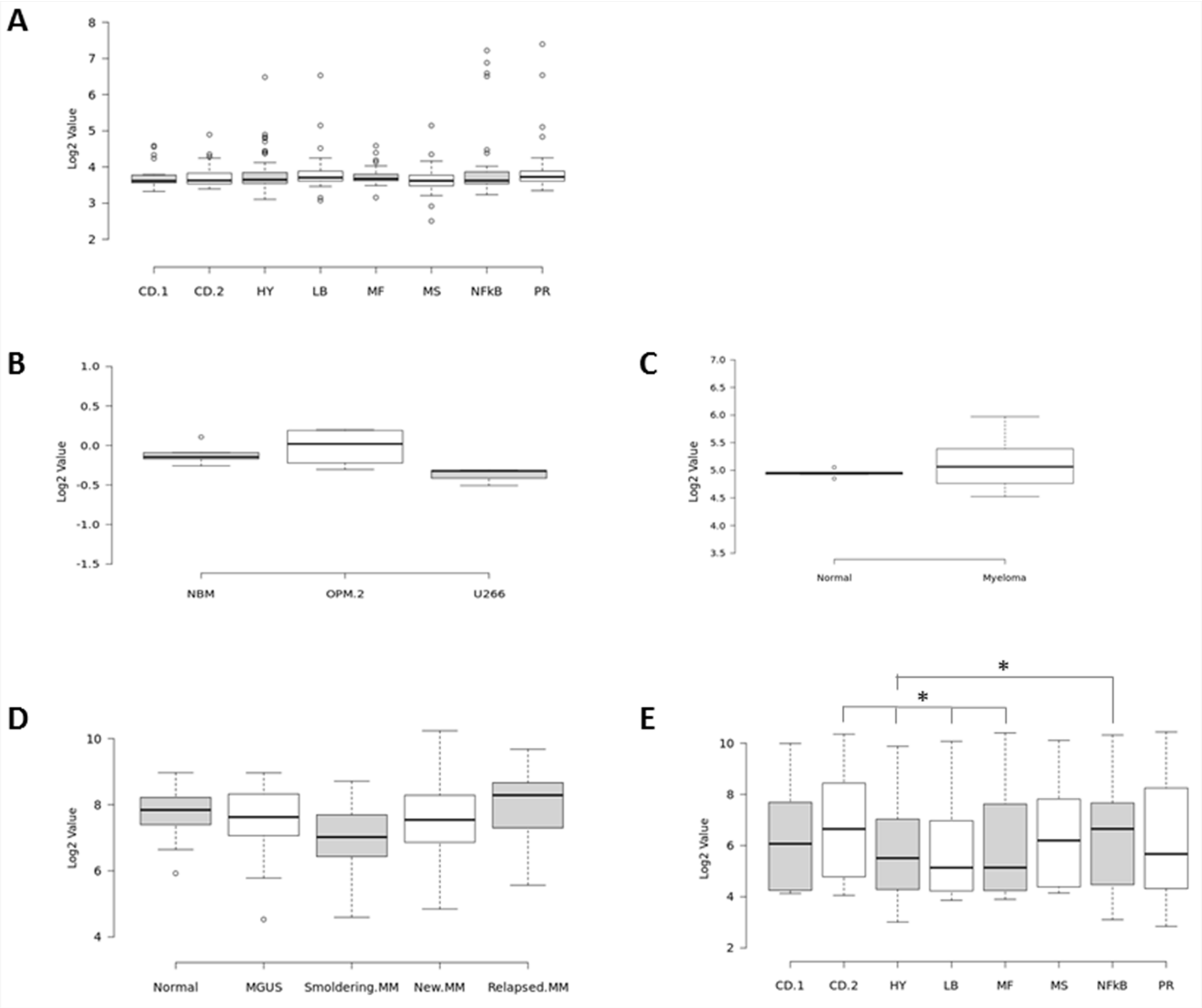

Supplementary Figure S2: **A.** FZR1 gene expression across molecular subgroups of MM; data from published dataset GSE19784 **B.** CDC20 gene expression in normal bone marrow (NBM), OPM-2 and U266 MM cell lines; GSE78884. **C.** CDC20 gene expression in CD138+ cells from normal donors and MM patients; data from published dataset GSE6477. **D.** CDC20 gene expression in CD138+ from normal donors, monoclonal gammopathy of undetermined significance (MGUS), smoldering MM, newly diagnosed MM and relapsed MM; data from published dataset GSE6691. **E.** CDC20 gene expression across molecular subgroups of MM; data from published dataset GSE19784. * $p \leq 0.05$ (t-test).

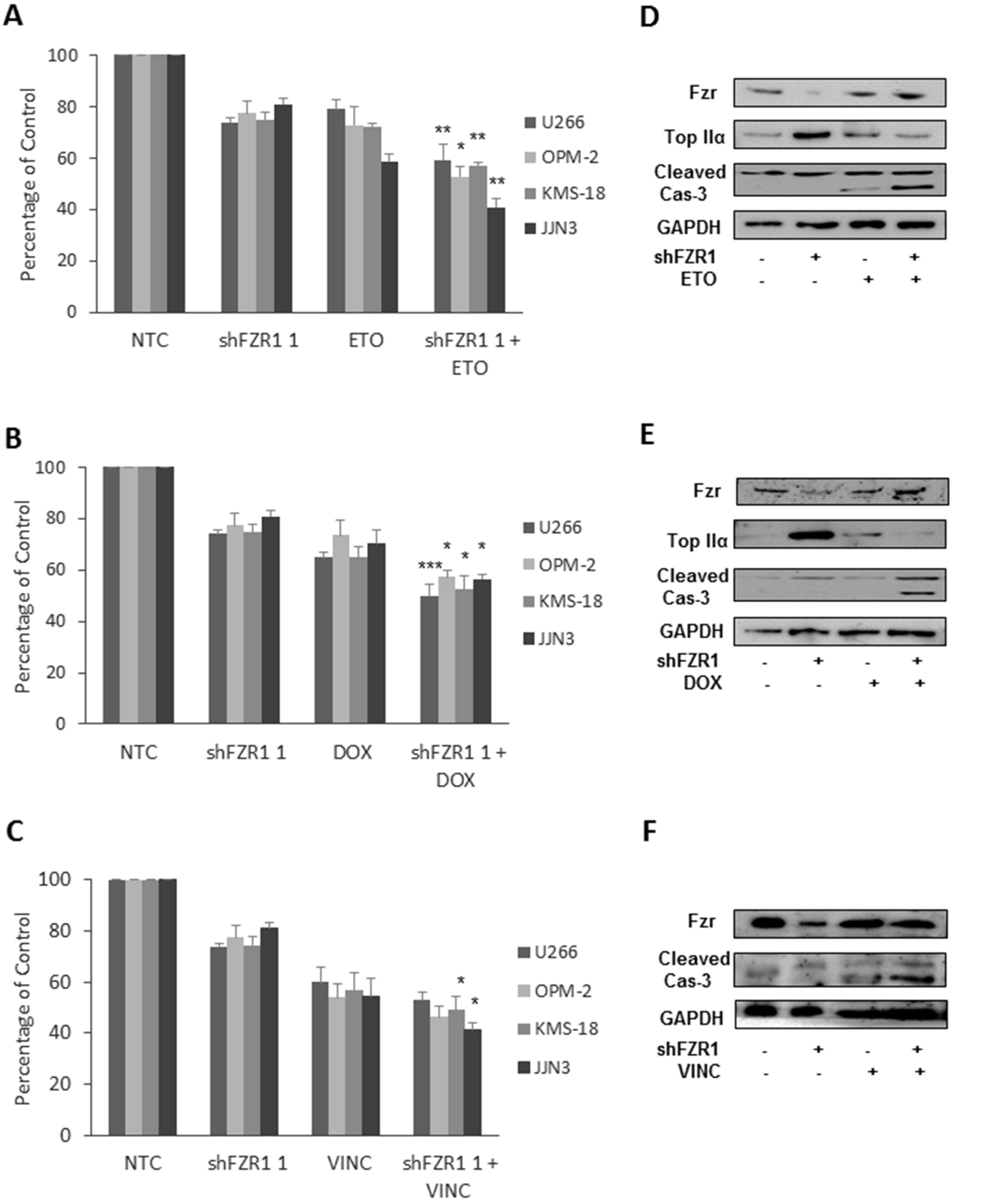

Supplementary Figure S3: FZR1 knockdown enhances the effect of conventional anti-mitotic agents in MM cell lines. **A-C.** MM cell lines were transduced with shFZR1 1 and after puromycin selection, NTC or shFZR1 cells were treated with 1µM etoposide (ETO), 100 nM doxorubicin (DOX), or 10 µM vincristine (VINC). Cell viability 48 hrs post-treatment; results shown are the mean of 3 independent experiments and are expressed as a percentage of vehicle treated control. **D-F.** Western blot analysis of Fzr knockdown, TopIIα and cleaved caspase-3 48 hrs post-treatment. * $p \leq 0.05$, ** $p \leq 0.01$, *** $p \leq 0.001$ (one-way ANOVA).

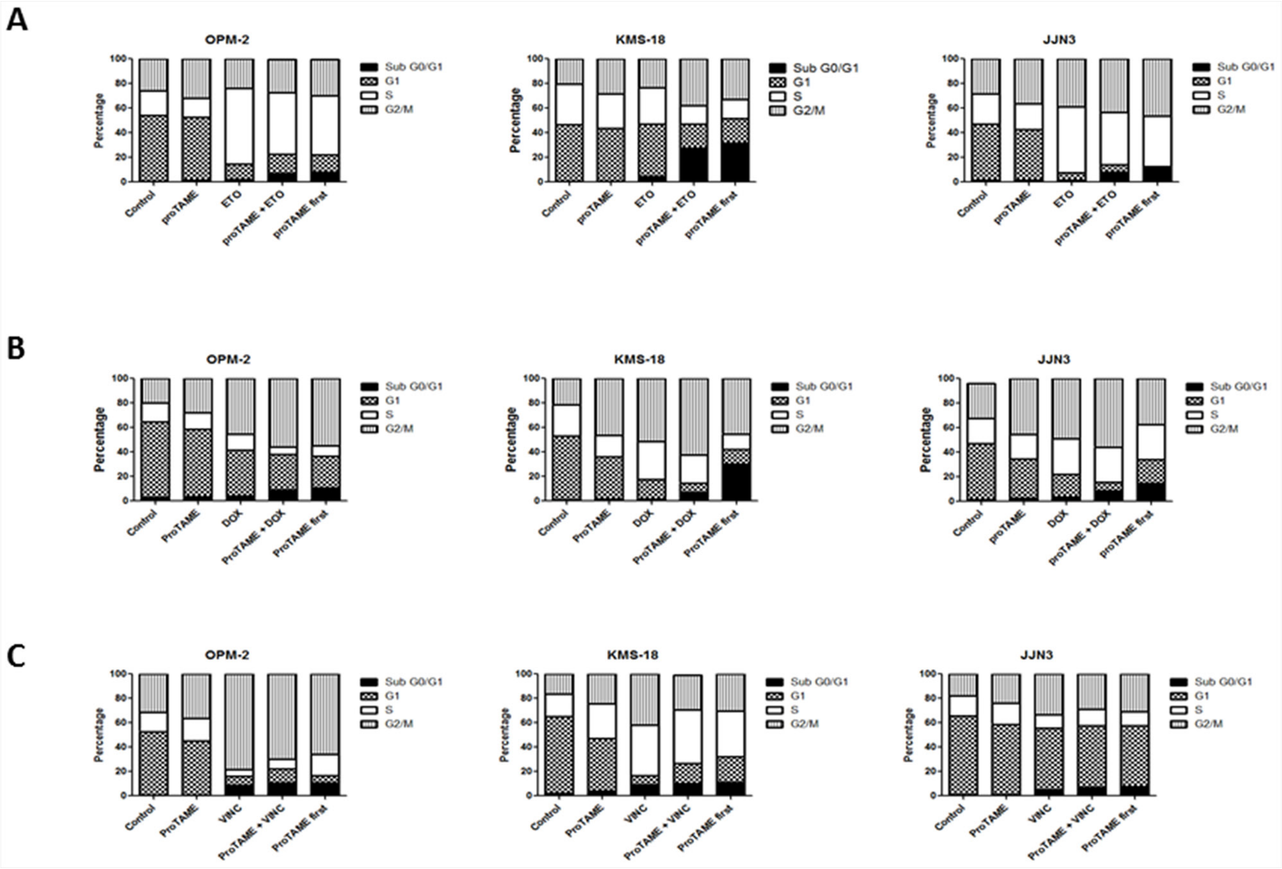

Supplementary Figure S4: A. Cell cycle analysis of MM cell lines treated with 10 µM proTAME, 1 µM etoposide (ETO) or a combination of both drugs added simultaneously or first pre-treated for 8 hrs with proTAME. **B.** Cell cycle analysis of MM cell lines treated with 10 µM proTAME, 100 nM doxorubicin (DOX) or a combination of both drugs added simultaneously or first pre-treated for 8 hrs with proTAME. **C.** Cell cycle analysis of MM cell lines treated with 10 µM proTAME, 10 µM vincristine (VINC) or a combination of both drugs added simultaneously or first pre-treated for 8 hrs with proTAME. Results shown represent a mean of 3 independent experiments.

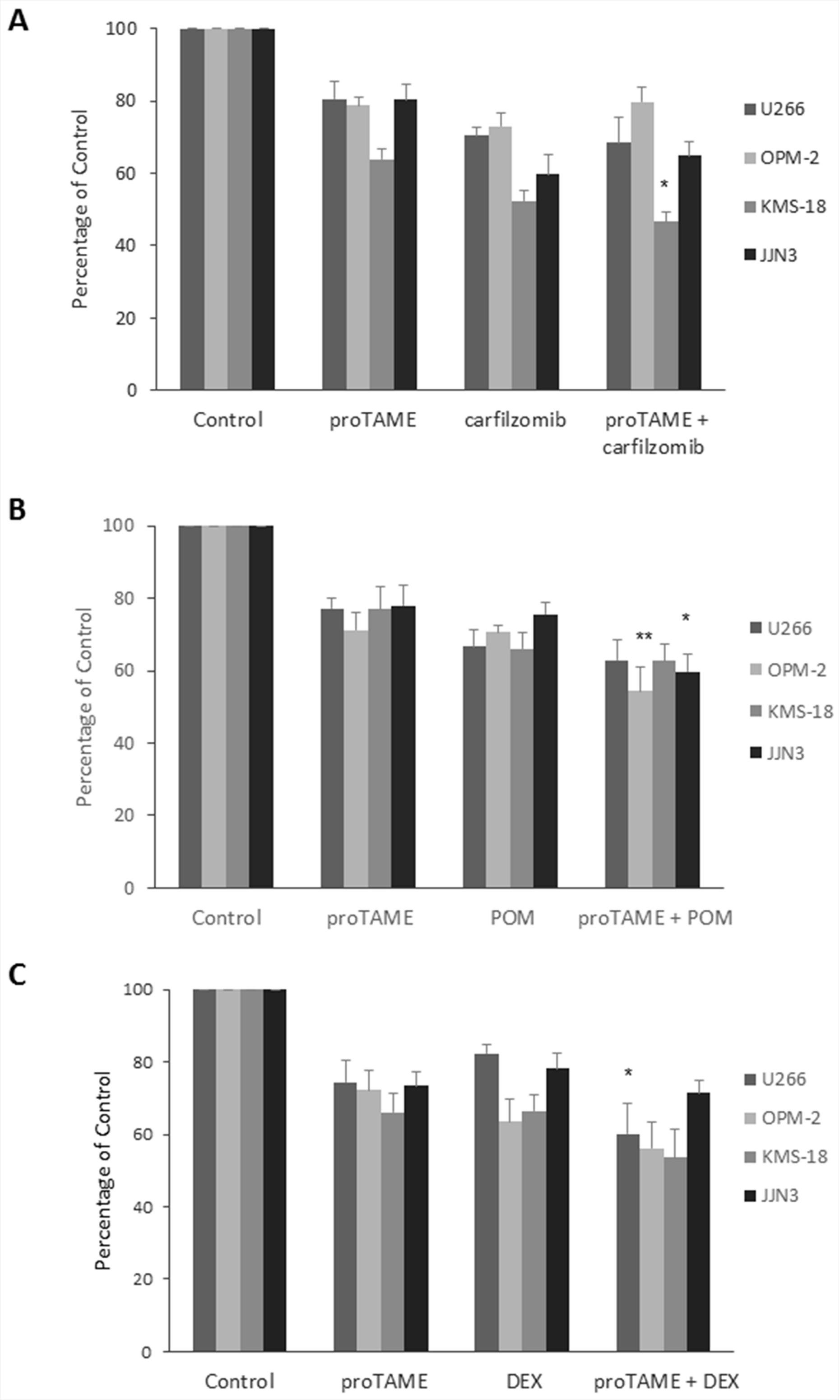

Supplementary Figure S5: A. MM cell lines were treated with 10 µM proTAME, 10 nM carfilzomib or a combination of both drugs added simultaneously. **B.** MM cell lines were treated with10 µM proTAME, 20 µM pomalidomide, or a combination of both drugs added simultaneously. **C.** MM cell lines were treated with10 µM proTAME, 20 nM dexamethasone, or a combination of both drugs added simultaneously. Cell viability 48 hrs post-treatment; results shown are the mean of 3 independent experiments and are expressed as a percentage of vehicle treated control. * $p \leq 0.05$, ** $p \leq 0.01$ (one-way ANOVA).
